# Protocols for Enzymatic Fluorometric Assays to Quantify Phospholipid Classes

**DOI:** 10.3390/ijms21031032

**Published:** 2020-02-04

**Authors:** Shin-ya Morita, Tokuji Tsuji, Tomohiro Terada

**Affiliations:** Department of Pharmacy, Shiga University of Medical Science Hospital, Otsu City, Shiga 520-2192, Japan

**Keywords:** enzymatic fluorometric measurement, phosphatidylcholine, phosphatidylethanolamine, phosphatidylserine, phosphatidic acid, phosphatidylinositol, phosphatidylglycerol, cardiolipin, sphingomyelin

## Abstract

Phospholipids, consisting of a hydrophilic head group and two hydrophobic acyl chains, are essential for the structures of cell membranes, plasma lipoproteins, biliary mixed micelles, pulmonary surfactants, and extracellular vesicles. Beyond their structural roles, phospholipids have important roles in numerous biological processes. Thus, abnormalities in the metabolism and transport of phospholipids are involved in many diseases, including dyslipidemia, atherosclerosis, cholestasis, drug-induced liver injury, neurological diseases, autoimmune diseases, respiratory diseases, myopathies, and cancers. To further clarify the physiological, pathological, and molecular mechanisms and to identify disease biomarkers, we have recently developed enzymatic fluorometric assays for quantifying all major phospholipid classes, phosphatidylcholine, phosphatidylethanolamine, phosphatidylserine, phosphatidic acid, phosphatidylinositol, phosphatidylglycerol + cardiolipin, and sphingomyelin. These assays are specific, sensitive, simple, and high-throughput, and will be applicable to cells, intracellular organelles, tissues, fluids, lipoproteins, and extracellular vesicles. In this review, we present the detailed protocols for the enzymatic fluorometric measurements of phospholipid classes in cultured cells.

## 1. Introduction

**Figure 1:**
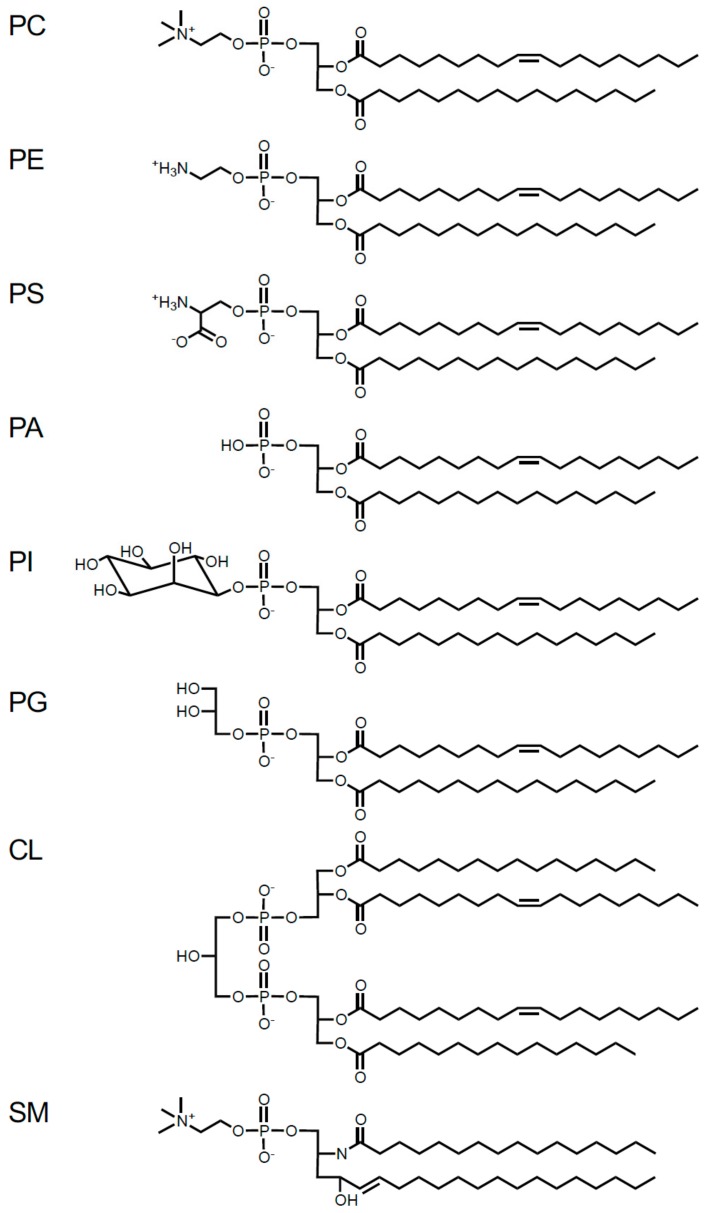
Structures of the phospholipid classes, phosphatidylcholine (PC), phosphatidylethanolamine (PE), phosphatidylserine (PS), phosphatidic acid (PA), phosphatidylinositol (PI), phosphatidylglycerol (PG), cardiolipin (CL), and sphingomyelin (SM). These phospholipid molecules contain various acyl chains.

Phospholipids are amphiphilic molecules composed of a hydrophilic head group and two hydrophobic acyl chains, and are divided into glycerophospholipids (GPL) containing a glycerol backbone and sphingophospholipids containing a sphingosine backbone. Glycerophospholipids are further divided based on the head group structures into the following classes: phosphatidylcholine (PC), phosphatidylethanolamine (PE), phosphatidylserine (PS), phosphatidic acid (PA), phosphatidylinositol (PI), phosphatidylglycerol (PG), and cardiolipin (CL) (Figure 1). Sphingomyelin (SM), a major sphingophospholipid class, has a phosphocholine moiety in the head group. Lysophospholipids, lysophosphatidylcholine (LPC), lysophosphatidylethanolamine (LPE), lysophosphatidylserine (LPS), lysophosphatidic acid (LPA), lysophosphatidylinositol (LPI), and lysophosphatidylglycerol (LPG), contain only one acyl chain. Sphingosylphosphorylcholine (SPC) is a lyso-form of SM. Furthermore, these phospholipids vary widely in the acyl chain composition.

It has been previously reported that abnormalities in phospholipid metabolism and transport are closely associated with disorders, including dyslipidemia, atherosclerosis, cholestasis, drug-induced liver injury, neurological diseases, autoimmune diseases, respiratory diseases, cardiac and skeletal myopathies, and cancers [1,2,3,4,5,6,7,8,9,10]. Therefore, phospholipid classes have potential as biomarkers for these diseases, and it is highly desired to develop high-sensitive and high-throughput methods for analyzing phospholipid classes.

The conventional assay for measuring phospholipid classes is thin-layer chromatography (TLC) followed by quantification of phosphate from the TLC spots [11]. However, this TLC-phosphorus assay is less sensitive, low throughput, time-consuming, and technically demanding. High-performance liquid chromatography (HPLC) equipped with an evaporative light-scattering detector or a corona charged aerosol detector has been used for the quantification of phospholipid classes [12,13,14]. Mass spectrometry has been applied to characterize phospholipid molecular species differing in acyl chain composition [15,16,17,18,19]. In mass spectrometric analyses, however, a large number of correction curves are needed for the quantification of each phospholipid molecular species because the ionization efficiencies differ among phospholipid species. In addition to these methods, we have recently completed the development of enzymatic fluorometric methods for quantifying all major phospholipid classes, which are specific, sensitive, simple, and high-throughput [18,20,21,22,23,24].

## 2. Physiological Roles of Phospholipids

### 2.1. Cell Membranes

Phospholipids, together with cholesterol, are necessary to assemble bilayer membranes in cells. In addition to the plasma membrane, the endoplasmic reticulum (ER), mitochondria, nuclear envelope, Golgi apparatus, lysosomes, endosomes, and peroxisomes are formed by bilayer membranes. In adipocytes, lipid droplets containing triglycerides are surrounded by phospholipid monolayers [25]. In mammalian cell membranes, the most abundant phospholipid is PC, constituting 40–50% of all phospholipids [26]. PE is the second most abundant mammalian phospholipid (20–50%), whereas PS, PA, PI, and PG are quantitatively minor components of cell membranes [26]. SM, together with cholesterol, is highly enriched in lipid raft membrane microdomains [27]. CL is located in the mitochondria, primarily in the inner mitochondrial membranes [28]. In addition to the structural roles in membranes, phospholipids play crucial roles in numerous cellular processes, including membrane protein localization and regulation, membrane trafficking, autophagy, cell proliferation and differentiation, apoptosis, cell migration, and intracellular signaling. For example, during apoptosis, PS is externalized to the cell surface as an ‘eat me’ signal that is recognized by phagocytes [29]. Microtubule-associated protein 1 light chain 3, an autophagy marker, is conjugated with PE [30]. Through direct interaction, PA positively regulates the mammalian target of rapamycin, which controls cell cycle progression and cell growth [31]. In mitochondria, CL facilitates electron transfer and ATP production [32]. PI is further phosphorylated to PI(4)P, PI(5)P, PI(3)P, PI(4,5)P_2_, PI(3,4)P_2_, PI(3,5)P_2_, and PI(3,4,5)P_3_, which are implicated in diverse cellular functions [7,33].

### 2.2. Plasma Lipoproteins

In the structures of plasma lipoproteins including high density lipoproteins (HDL), low density lipoproteins (LDL), very low density lipoproteins (VLDL), and chylomicrons, a hydrophobic core consisting of triglyceride and cholesteryl esters is enveloped by a surface monolayer consisting of phospholipids, cholesterol, and apolipoproteins [6]. In the formation of VLDL in hepatocytes and chylomicrons in enterocytes, the microsomal triglyceride transfer protein mediates the transfer of phospholipids from the endoplasmic reticulum membrane or another site to apolipoprotein B [6]. HDL is formed from cellular phospholipids and cholesterol through the interaction of apolipoprotein A-I with the ATP-binding cassette (ABC) transporter ABCA1 at the cell surface plasma membrane [6,34,35]. PC and SM are the main phospholipids in lipoprotein particles. The SM/PC ratio is ~0.25 in VLDL particles but increases to ~0.5 in LDL particles, because the degradation of SM is much slower than that of PC in plasma [6]. The PC/SM ratio in HDL is ~0.2 [36]. The metabolism of lipoproteins is highly dependent on the composition of surface phospholipids through the regulation of binding of apolipoproteins or enzymes on the lipoprotein surfaces [6,37,38,39]. For example, SM at the lipid particle surface reduces the binding of apolipoprotein E to the particle [37]. Apolipoprotein E on the lipoprotein surface also binds to the LDL receptor, LDL receptor-related protein and heparan sulfate proteoglycans, which promotes the uptake of lipoprotein remnant particles by hepatocytes [6]. However, little is known about the roles of other phospholipid classes on the lipoprotein particle surfaces.

### 2.3. Biliary Mixed Micelles

In bile, bile salts, phospholipids, and cholesterol are the main lipid components, which form mixed micelles and vesicles. Bile salts damage cell membranes due to their detergent properties. The biliary phospholipids are necessary to protect hepatocytes from bile salt cytotoxicity [8,40]. The cytotoxicity of bile salts is attenuated by the formation of mixed micelles with phospholipids. PC is the predominant (>95%) biliary phospholipid, whereas only small amounts of PE, PS, and SM are present in bile [8,41]. The phospholipid secretion from hepatocytes into bile is mediated by the transporter ABCB4 [41,42,43,44]. *ABCB4* gene mutations cause a wide spectrum of liver diseases, including progressive familial intrahepatic cholestasis type 3, intrahepatic cholestasis of pregnancy, low phospholipid-associated cholelithiasis, primary biliary cirrhosis, and cholangiocarcinoma [8,41].

### 2.4. Pulmonary Surfactants

Pulmonary surfactants, extracellular complex of phospholipids, cholesterol and proteins, are secreted by type II alveolar epithelial cells, and reduce the incidence of alveoli to collapse during expiration [45]. The dysfunction of pulmonary surfactants disturbs alveolar gas exchange. In pulmonary surfactants, PC is the most abundant phospholipid class (70–80%), and 1,2-dipalmitoyl PC is the most abundant molecular species [45,46]. The second most abundant phospholipid is PG, constituting up to 10%, and PI, PE, and PS are also present in the surfactants [45,46]. PG and PI in pulmonary surfactants play key roles in regulating inflammatory processes within the lung [46]. In type II cells, the ABCA3 transporter protein is located at the limiting membrane of lamellar bodies, and plays an essential role in the biogenesis of lamellar bodies, likely by transporting phospholipids [3,47]. Phospholipids and proteins contained in the lamellar body are secreted into the alveoli and form films preventing alveolar collapse [3]. The majority of *ABCA3* mutations result in neonatal respiratory distress syndrome [3]. 

### 2.5. Extracellular Vesicles

Extracellular vesicles are secreted by almost all types of cells, and widely observed in body fluids, including blood, urine, milk, saliva, cerebrospinal fluid, and semen [48,49]. Extracellular vesicles consist of phospholipid bilayer membranes with transmembrane proteins and contain microRNAs, mRNAs, DNAs, and proteins [48,49]. Extracellular vesicles are categorized into three groups, exosomes (30–150 nm in diameter), microvesicles (100–1000 nm) and apoptotic bodies (1–5 µm) [48,49]. Microvesicles and exosomes are generated from plasma membranes and endosomal compartments, respectively [48,49,50]. Multivesicular bodies filled with vesicles originate from endosomes and fuse with the plasma membranes to release exosomes into the extracellular space [48,49,50]. Recently, an increasing number of studies have demonstrated that extracellular vesicles play important roles in intracellular communication and many pathophysiologies [48,49,50,51]. In particular, extracellular vesicles from cancer cells target diverse types of normal cells to alter the microenvironment in order to support the cancer cell growth [48,49,51]. Thus, extracellular vesicles hold great promise as biomarkers. On the other hand, the roles of phospholipids in the bioactivities and fates of extracellular vesicles remain largely unknown, although several studies have analyzed the phospholipid composition of exosomes [51,52].

## 3. Phospholipid Biomarkers

As the level of serum phospholipids increases in patients with obstructive jaundice, the measurement of choline-containing phospholipids in serum is used as a clinical laboratory test to diagnose liver diseases [53,54]. Lipoprotein-X (Lp-X) is detected in the plasma of patients with extrahepatic and intrahepatic cholestasis caused by bile duct obstruction, hepatitis, primary sclerosing cholangitis, or primary biliary cholangitis [9,55,56]. Moreover, in patients with drug-induced cholestasis, plasma phospholipid and Lp-X levels markedly increase [1,57]. Lp-X is an abnormal lipoprotein rich in phospholipids and cholesterol, and is observed as vesicles of 50–70 nm in diameter [9]. The biliary phospholipid secretion mediated by Abcb4 is required for the appearance of Lp-X in bile duct-ligated mice, suggesting that Lp-X originates from biliary phospholipids moving into blood vessels under cholestatic conditions [9,58].

In addition to high levels of LDL-cholesterol and low levels of HDL-cholesterol, high plasma SM levels have been found to be an independent risk factor for coronary artery disease after adjusting for other risk factors [59]. Of note, LDL extracted from human atherosclerotic lesions exhibits a higher ratio of SM/PC than plasma LDL [60]. In arterial walls, sphingomyelinase (SMase) converts SM to ceramide in LDL, which leads to LDL aggregation and subsequent macrophage foam cell formation [6,38,39,61].

## 4. Detection of H_2_O_2_ in Enzymatic Assays

Our developed assays for measuring phospholipid classes (described in Section 6) fluorometrically detect enzymatically produced H_2_O_2_ in the final steps. This section describes several probes for detecting H_2_O_2_.

**Figure 2:**
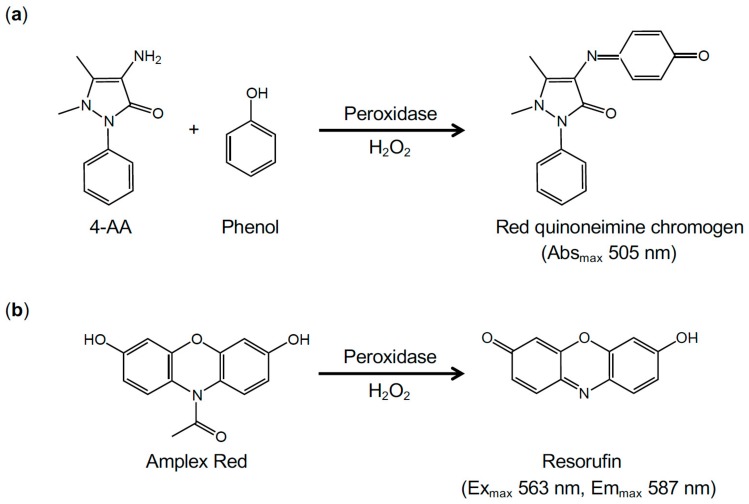
Detection of H_2_O_2_ in enzymatic assays. (**a**) The oxidative coupling of phenol and 4-AA by H_2_O_2_ in the presence of peroxidase produces a red quinoneimine chromogen (absorption maximum at 505 nm). (**b**) The oxidation of Amplex Red by H_2_O_2_ in the presence of peroxidase produces highly fluorescent resorufin (excitation maximum at 563 nm and emission maximum at 587 nm).

In the final steps of many enzymatic assays, enzymatically generated H_2_O_2_ is determined spectrophotometrically by reaction with chromogenic hydrogen donors catalyzed by peroxidase. As shown in Figure 2a, the oxidative condensation of phenol with 4-amino antipyrine (4-AA) by H_2_O_2_ in the presence of peroxidase yields a red quinoneimine chromogen with an absorption maximum at 505 nm, which has been widely used in clinical laboratory tests [53,62,63]. *N*-Ethyl-*N*-(2-hydroxy-3-sulfopropyl)-3,5-dimethoxyaniline (DAOS) and *N*-ethyl-*N*-(2-hydroxy-3-sulfopropyl)-3-methylaniline (TOOS) are also oxidatively coupled with 4-AA by H_2_O_2_, and these resulting chromogens have higher molar extinction coefficients and longer wavelengths of maximum absorption (593 nm and 555 nm, respectively) than the chromogen produced by the coupling of phenol and 4-AA [64].

4-Hydroxyphenyl compounds, homovanillic acid, 4-hydroxyphenylacetic acid and 3-(4-hydroxyphenyl)propionic acid (HPPA), are fluorogenic substrates for peroxidase and have been employed for the H_2_O_2_ assay [65,66,67]. Using HPPA, H_2_O_2_ can be measured at as low as 100 pmol [67]. In the presence of H_2_O_2_, peroxidase catalyzes the dimerization of HPPA to form fluorescent bisphenol with excitation and emission maxima of 318 nm and 404 nm, respectively [68].

10-Acetyl-3,7-dihydroxyphenoxazine (Amplex Red) is also applied for monitoring of H_2_O_2_ levels and in various enzymatic assays [69,70,71,72,73]. Peroxidase-catalyzed oxidation of Amplex Red, a nonfluorescent compound, by H_2_O_2_ generates highly fluorescent resorufin with an excitation maximum at 563 nm and emission maximum at 587 nm [69] (Figure 2b). The reaction stoichiometry of Amplex Red and H_2_O_2_ is 1:1. Amplex Red can detect as little as 2 pmol of H_2_O_2_ [71].

## 5. Enzymatic Assays of Phospholipids

Takayama et al. have developed the first enzymatic method to measure choline-containing phospholipids, PC, LPC, and SM, which involves three steps [53]. (1) PC, LPC, or SM is hydrolyzed by phospholipase D (PLD) from *Streptomyces* sp. to release choline. (2) Choline is oxidized by choline oxidase to betaine, which simultaneously generates H_2_O_2_. (3) In the presence of peroxidase, H_2_O_2_ couples phenol and 4-AA to produce a chromogen. This method does not distinguish among PC, LPC, and SM.

Enzymatic assays have been also reported for the specific measurement of SM by Blaton et al. [54]. In this assay, SM is selectively hydrolyzed by SMase from *Bacillus cereus* to liberate phosphocholine. Next, alkaline phosphatase catalyzes the cleavage of inorganic phosphate and the formation of choline. Choline is oxidized by choline oxidase, and the generated H_2_O_2_ is subsequently detected by phenol and 4-AA in the presence of peroxidase.

Hojjati and Jiang have reported an enzymatic method for the specific quantification of PC [74]. In the first step of this method, GPL-specific PLD (GPL-PLD) hydrolyzes PC, but not SM, to choline and PA. Choline is oxidized in a reaction catalyzed by choline oxidase to generate H_2_O_2_, which induces the coupling of DAOS and 4-AA in the presence of peroxidase as catalyst to yield a blue dye.

Ota et al. have reported the preliminary application of amine oxidase from *Arthrobacter* sp. to measure PE [75]. This PE assay consists of the hydrolysis of PE by PLD, oxidative deamination of ethanolamine by amine oxidase to generate H_2_O_2_, and oxidative coupling reaction between TOOS and 4-AA catalyzed by peroxidase.

## 6. Enzymatic Fluorometric Assays for Quantifying Phospholipid Classes

To enable comprehensive quantification of all major phospholipid classes in mammalian cells, we have developed methods for measuring PC, PE, PS, PA, PI, PG + CL, and SM using combinations of specific enzymes and Amplex Red [18,20,21,22,23,24]. This section describes the protocols for the enzymatic fluorometric assays for quantifying these phospholipid classes in cultured cells and intracellular organelles, which are slightly modified from those in the original reports mainly due to the availability of commercial enzymes (GPL-PLD for PC assay and glycerol-3-phosphate (G3P) oxidase for PA and PG + CL assays).

### 6.1. Sample Preparation

To determine the cellular phospholipid contents, cells are cultured in 10-cm dishes or 6-well plates. Cells were washed, scraped with cold phosphate-buffered saline, and sonicated to prepare cell homogenates. To determine the phospholipid contents in purified mitochondrial and microsomal fractions, cells are cultured in 10-cm dishes. Purified mitochondrial and microsomal fractions are isolated according to the previously described method [76]. The concentrations of protein in samples are measured by the bicinchoninic acid assay [77]. Phospholipids in cell homogenates or in purified mitochondrial and microsomal fractions are extracted by the modified method of Folch [17,24,78,79]. Lipid extraction from samples is recommended for the enzymatic measurements of phospholipid classes to remove water-soluble interfering compounds including choline, amines, amino acids, G3P, inositol, NADH, and H_2_O_2_.

### 6.2. Lipid Extraction

#### 6.2.1. Materials

- Chloroform (≥99.0%) (08402-55, Nacalai Tesque, Kyoto, Japan).
- Methanol (≥99.8%) (21915-35, Nacalai Tesque).
- Triton X-100 (<1 ppm H_2_O_2_) (10789704001, Roche Diagnostics, Mannheim, Germany).

#### 6.2.2. Procedure

1. Pipette each sample (1 mL) into a glass tube.
2. Add chloroform/methanol (2:1) solution (4 mL) to each glass tube and vortex.
3. Incubate overnight at 4 °C.
4. Complete the phase split by centrifugation (740× *g*, 20 min, 4 °C).
5. Carefully remove the upper aqueous phase and the interfacial material using a glass Pasteur pipette.
6. Add H_2_O (1 mL) to the recovered lower organic phase and vortex.
7. Complete the phase split by centrifugation and remove the aqueous phase again.
8. Evaporate the organic solvent from the lower phase.
9. Dissolve the evaporated sample with 1% Triton X-100 solution (200 μL).

### 6.3. Protocol for Enzymatic Fluorometric Measurement of PC

#### 6.3.1. Strategy

**Figure 3:**
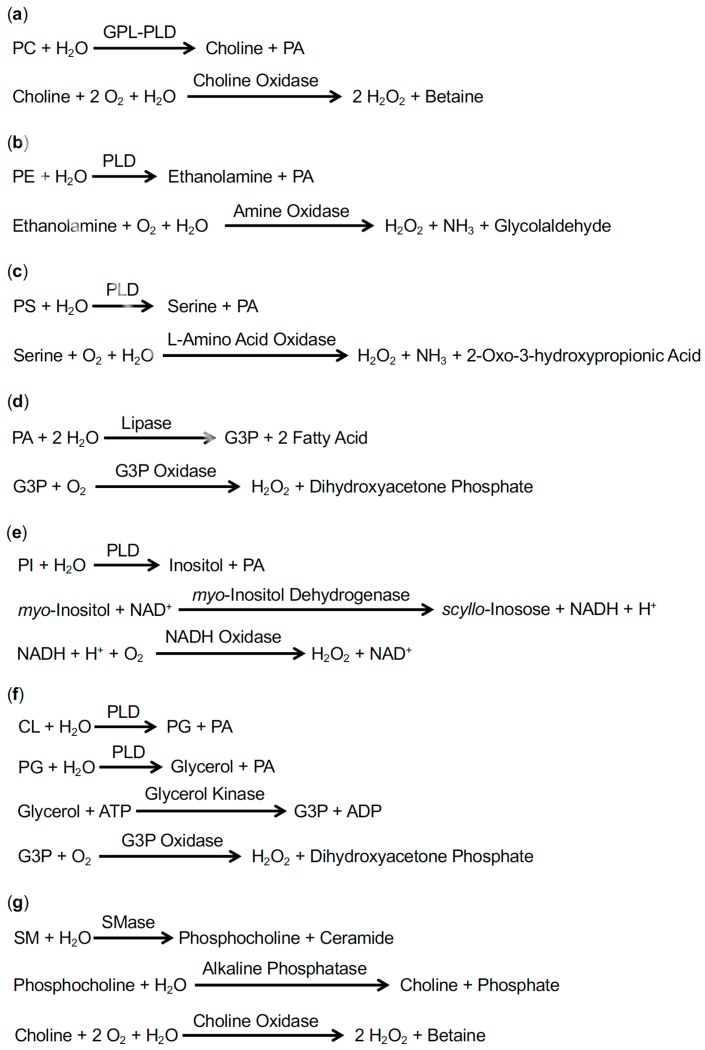
Strategies for enzymatic fluorometric measurements of PC (**a**), PE (**b**), PS (**c**), PA (**d**), PI (**e**), PG + CL (**f**), and SM (**g**). In the final steps, H_2_O_2_ is detected using Amplex Red and peroxidase.

There are three reaction steps for the enzymatic fluorometric measurement of PC [18] (Figure 3a).

1. PC is hydrolyzed by GPL-PLD to choline and PA.
2. Choline is oxidized by choline oxidase, generating two H_2_O_2_ molecules and betaine.
3. In the presence of peroxidase, H_2_O_2_ reacts with Amplex Red to produce resorufin.

#### 6.3.2. Materials

- GPL-PLD from *Streptomyces* sp. (T-39, Asahi Kasei Pharma, Tokyo, Japan).
- Choline oxidase from *Alcaligenes* sp. (037-14401, FUJIFILM Wako Pure Chemical, Osaka, Japan).
- Peroxidase from horseradish roots (46261003, Oriental Yeast, Tokyo, Japan).
- Amplex Red (A12222, Thermo Fisher Scientific, Waltham, MA, USA).
- Amplex Red/UltraRed Stop Reagent (A33855, Thermo Fisher Scientific).
- Egg PC (l-α-PC from chicken egg) (840051P, Avanti Polar Lipids, Alabaster, AL, USA).
- 96-well black flatbottom plate S type for fluorescence measurements (MS-8496K, Sumitomo Bakelite, Tokyo, Japan).
- Triton X-100 (see Section 6.2.1).

#### 6.3.3. Reagents

- Reagent C1: 100 U/mL GPL-PLD, 1.5 mM CaCl_2_, 50 mM NaCl, and 50 mM Tris-HCl (pH 7.4).
- Reagent C2: 4 U/mL choline oxidase, 5 U/mL peroxidase, 300 µM Amplex Red, 0.2% Triton X-100, 50 mM NaCl, and 50 mM Tris-HCl (pH 7.4).
- Solubilize egg PC (average M.W. 770.12) at 25 mM in 10% Triton X-100 aqueous solution, and then dilute with water to 2.5 mM in 1% Triton X-100 solution. To prepare PC standard solutions, 2.5 mM egg PC in 1% Triton X-100 is sequentially diluted with 1% Triton X-100 solution. The 25 mM egg PC in 10% Triton X-100 solution is stored at −20 °C.

#### 6.3.4. Procedure

1. Pipette each sample or PC standard solution (10 µL) into a 96-well black plate.
2. Add Reagent C1 (40 µL) to each well and incubate at 37 °C for 30 min.
3. Add Reagent C2 (50 µL) to each well and incubate at room temperature for 30 min.
4. Add Amplex Red Stop Reagent (20 µL) to each well.
5. Measure the fluorescence intensity at 544 nm (excitation) and 590 nm (emission) using a microplate reader (Infinite M200, Tecan, Männedorf, Switzerland).

#### 6.3.5. Sensitivity and Specificity

The standard curve for the PC measurement is quadratic at concentrations below 20 μM and linear between 20 and 150 μM [18]. The detection limit is 1 µM (10 pmol in the reaction mixture). There are no differences in the fluorescence changes in response to egg PC, soy PC, 1-palmitoyl-2-oleoyl PC, and plasmanylcholine, indicating that this PC measurement is not affected by the chain length, the number of double bonds or the linkage type (ester or ether). In this PC assay, other choline-containing phospholipids, SM, and LPC, induce only negligible increases in fluorescence. In the recovery test using the cellular lipid extract, the mean recovery of 1-palmitoyl-2-oleoyl PC in concentrations of 12.5–75.0 μM is 101.3%, indicating that there is no interference of hydrophobic compounds extracted from the cells [18].

### 6.4. Protocol for Enzymatic Fluorometric Measurement of PE

#### 6.4.1. Strategy

There are three reaction steps for the enzymatic fluorometric measurement of PE [18] (Figure 3b).

1. PE is hydrolyzed by PLD to ethanolamine and PA.
2. Ethanolamine is oxidized by amine oxidase, generating H_2_O_2_, NH_3_ and glycolaldehyde.
3. In the presence of peroxidase, H_2_O_2_ reacts with Amplex Red to produce resorufin.

#### 6.4.2. Materials

- PLD from *Streptomyces chromofuscus* (BML-SE301, Enzo Life Sciences, Farmingdale, NY, USA).
- Amine oxidase (tyramine oxidase) from *Arthrobacter* sp. (T-25, Asahi Kasei Pharma).
- Liver PE (l-α-PE from bovine liver) (840026P, Avanti Polar Lipids).
- Triton X-100 (see Section 6.2.1). Peroxidase, Amplex Red, Amplex Red/UltraRed Stop Reagent, and 96-well black flatbottom plate (see Section 6.3.2).

#### 6.4.3. Reagents

- Reagent E1: 150 U/mL PLD, 1.5 mM CaCl_2_, 50 mM NaCl, and 50 mM Tris-HCl (pH 7.4).
- Reagent E2: 8 U/mL amine oxidase, 5 U/mL peroxidase, 300 µM Amplex Red, 0.2% Triton X-100, 50 mM NaCl, and 50 mM Tris-HCl (pH 7.4).
- Solubilize liver PE (average M.W. 756.34) at 25 mM in 10% Triton X-100 aqueous solution, and then dilute with water to 2.5 mM in 1% Triton X-100 solution. To prepare PE standard solutions, 2.5 mM liver PE in 1% Triton X-100 is sequentially diluted with 1% Triton X-100 solution. The 25 mM liver PE in 10% Triton X-100 solution is stored at −20 °C.

#### 6.4.4. Procedure

1. Pipette each sample or PE standard solution (10 µL) into a 96-well black plate.
2. Add Reagent E1 (40 µL) to each well and incubate at 37 °C for 30 min.
3. Add Reagent E2 (50 µL) to each well and incubate at room temperature for 30 min.
4. Add Amplex Red Stop Reagent (20 µL) to each well.
5. Measure the fluorescence intensity at 544 nm (excitation) and 590 nm (emission) using a microplate reader.

#### 6.4.5. Sensitivity and Specificity

The standard curve for the PE measurement is quadratic at concentrations below 50 μM and linear between 50 and 250 μM [18]. The detection limit is 1 µM (10 pmol in the reaction mixture). There are no differences in the fluorescence changes in response to liver PE, soy PE, 1-palmitoyl-2-oleoyl PE, plasmenylethanolamine, and LPE, indicating that this PE measurement is not affected by the chain length, the number of double bonds, or the linkage type (ester or ether), and does not distinguish between PE and LPE. In this PE assay, other amine-containing phospholipids, PC and PS, lead to no increase in fluorescence. In the recovery test using the cellular lipid extract, the mean recovery of 1-palmitoyl-2-oleoyl PE in concentrations of 12.5–75.0 μM is 99.6% [18].

### 6.5. Protocol for Enzymatic Fluorometric Measurement of PS

#### 6.5.1. Strategy

There are three reaction steps for the enzymatic fluorometric measurement of PS [21] (Figure 3c).

1. PS is hydrolyzed by PLD to serine and PA.
2. Serine is oxidized by l-amino acid oxidase, generating H_2_O_2_ and 2-oxo-3-hydroxypropionic acid.
3. In the presence of peroxidase, H_2_O_2_ reacts with Amplex Red to produce resorufin.

#### 6.5.2. Materials

- PLD from *Streptomyces chromofuscus* (BML-SE301, Enzo Life Sciences).
- L-Amino acid oxidase from *Crotalus adamanteus* venom (LAO, Worthington Biochemical, Lakewood, NJ, USA).
- Brain PS (l-α-PS sodium salt from porcine brain) (840032P, Avanti Polar Lipids).
- Triton X-100 (see Section 6.2.1). Peroxidase, Amplex Red, Amplex Red/UltraRed Stop Reagent, and 96-well black flatbottom plate (see Section 6.3.2).

#### 6.5.3. Reagents

- Reagent S1: 600 U/mL PLD, 25 U/mL l-amino acid oxidase, 50 mM NaCl, and 50 mM Tris-HCl (pH 7.4).
- Reagent S2: 6.25 U/mL peroxidase, 187.5 µM Amplex Red, 0.125% Triton X-100, 50 mM NaCl, and 50 mM Tris-HCl (pH 7.4).
- Solubilize brain PS (average M.W. 824.97) at 25 mM in 10% Triton X-100 aqueous solution, and then dilute with water to 2.5 mM in 1% Triton X-100 solution. To prepare PS standard solutions, 2.5 mM brain PS in 1% Triton X-100 is sequentially diluted with 1% Triton X-100 solution. The 25 mM brain PS in 10% Triton X-100 solution is stored at −20 °C.

#### 6.5.4. Procedure

1. Pipette each sample or PS standard solution (10 µL) into a 96-well black plate.
2. Add Reagent S1 (10 µL) to each well and incubate at 25 °C for 240 min.
3. Add Reagent S2 (80 µL) to each well and incubate at room temperature for 15 min.
4. Add Amplex Red Stop Reagent (20 µL) to each well.
5. Measure the fluorescence intensity at 544 nm (excitation) and 590 nm (emission) using a microplate reader.

#### 6.5.5. Sensitivity and Specificity

The standard curve for the PS measurement is linear at concentrations below 50 μM and hyperbolic between 50 and 1000 μM [21]. The detection limit is 5 µM (50 pmol in the reaction mixture). There are no differences in the fluorescence changes in response to brain PS, soy PS, 1-palmitoyl-2-oleoyl PS, and LPS, indicating that this PS measurement is not affected by the chain length or the number of double bonds, and does not distinguish between PS and LPS. In this PS assay, other amine-containing phospholipids, PC and PE, lead to no increase in fluorescence. In the recovery test using the cellular lipid extract, the mean recovery of 1-palmitoyl-2-oleoyl PS in concentrations of 25–125 μM is 98.6% [21]. This PS enzymatic fluorometric assay and TLC-phosphorus assay correlate well [21].

### 6.6. Protocol for Enzymatic Fluorometric Measurement of PA

#### 6.6.1. Strategy

There are three reaction steps for the enzymatic fluorometric measurement of PA [20] (Figure 3d).

1. PA is hydrolyzed by lipase to G3P and two fatty acids.
2. G3P is oxidized by G3P oxidase, generating H_2_O_2_ and dihydroxyacetone phosphate.
3. In the presence of peroxidase, H_2_O_2_ reacts with Amplex Red to produce resorufin.

#### 6.6.2. Materials

- Lipase (lipoprotein lipase) from *Pseudomonas* sp. (129-04501, FUJIFILM Wako Pure Chemical).
- G3P oxidase (l-α-glycerophosphate oxidase) from *Streptococcus* sp. (T-60, Asahi Kasei Pharma).
- Egg PA (l-α-PA sodium salt from chicken egg) (840101P, Avanti Polar Lipids).
- The 0.2-ml PCR tube with a flat cap (FG-021F, NIPPON Genetics, Tokyo, Japan).
- Triton X-100 (see Section 6.2.1). Peroxidase, Amplex Red, Amplex Red/UltraRed Stop Reagent, and 96-well black flatbottom plate (see Section 6.3.2).

#### 6.6.3. Reagents

- Reagent A1: 40,000 U/mL lipase, 50 mM NaCl, and 50 mM Tris-HCl (pH 7.4).
- Reagent A2: 10 U/mL G3P oxidase, 5 U/mL peroxidase, 300 µM Amplex Red, 0.2% Triton X-100, 50 mM NaCl, and 50 mM Tris-HCl (pH 7.4).
- Solubilize egg PA (average M.W. 706.16) at 25 mM in 10% Triton X-100 aqueous solution, and then dilute with water to 2.5 mM in 1% Triton X-100 solution. To prepare PA standard solutions, 2.5 mM egg PA in 1% Triton X-100 is sequentially diluted with 1% Triton X-100 solution. The 25 mM egg PA in 10% Triton X-100 solution is stored at −20 °C.

#### 6.6.4. Procedure

1. Pipette each sample or PA standard solution (20 µL) into a 0.2-ml tube.
2. Add Reagent A1 (80 µL) to each tube, incubate at 37 °C for 30 min, and then heat at 96 °C for 3 min using a thermal cycler.
3. Precipitate the denatured enzyme by centrifugation (7200× *g*, 5 min, room temperature).
4. Pipette the supernatant (50 µL) into a 96-well black plate.
5. Add Reagent A2 (50 µL) to each well and incubate at room temperature for 30 min.
6. Add Amplex Red Stop Reagent (20 µL) to each well.
7. Measure the fluorescence intensity at 544 nm (excitation) and 590 nm (emission) using a microplate reader.

#### 6.6.5. Sensitivity and Specificity

The standard curve for the PA measurement is quadratic at concentrations below 50 μM and linear between 50 and 250 μM [20]. The detection limit is 5 µM (50 pmol in the reaction mixture). There are no differences in the fluorescence changes in response to egg PA and LPA, indicating that this PA measurement is not affected by the chain length or the number of double bonds, and does not distinguish between PA and LPA.

### 6.7. Protocol for Enzymatic Fluorometric Measurement of PI

#### 6.7.1. Strategy

There are four reaction steps for the enzymatic fluorometric measurement of PI [24] (Figure 3e).

1. PI is hydrolyzed by PLD to *myo*-inositol and PA.
2. The oxidation of *myo*-inositol and the reduction of NAD^+^ are catalyzed by *myo*-inositol dehydrogenase, generating *scyllo*-inosose and NADH, respectively.
3. NADH is oxidized by NADH oxidase, generating H_2_O_2_ and NAD^+^.
4. In the presence of peroxidase, H_2_O_2_ reacts with Amplex Red to produce resorufin.

#### 6.7.2. Materials

- PLD from *Streptomyces chromofuscus* (T-222, Asahi Kasei Pharma).
- *myo*-Inositol dehydrogenase from *Bacillus subtilis* (E-INDHBS, Megazyme, Bray, Ireland).
- NADH oxidase from *Bacillus licheniformis* (23626-52, Nacalai Tesque, Kyoto, Japan).
- NAD^+^ (β-nicotinamide adenine dinucleotide) (24338-31, Nacalai Tesque).
- Liver PI (L-α-PI sodium salt from bovine liver) (840042P, Avanti Polar Lipids).
- Triton X-100 (see Section 6.2.1). Peroxidase, Amplex Red, Amplex Red/UltraRed Stop Reagent, and 96-well black flatbottom plate (see Section 6.3.2). 0.2-mL tube (see Section 6.6.2).

#### 6.7.3. Reagents

- Reagent I1: 200 units/mL PLD, 2.4 mM CaCl_2_, 50 mM NaCl, and 50 mM Tris-HCl (pH 7.4).
- Reagent I2: 25 units/mL *myo*-inositol dehydrogenase, 10 mM NAD^+^, 150 mM NaCl, and 150 mM Tris-HCl (pH 7.4).
- Reagent I3: 1 U/mL NADH oxidase, 6.25 units/mL peroxidase, 187.5 µM Amplex Red, 0.125% Triton X-100, 50 mM NaCl, and 50 mM Tris-HCl (pH 7.4).
- Solubilize liver PI (average M.W. 902.13) at 25 mM in 10% Triton X-100 aqueous solution, and then dilute with water to 2.5 mM in 1% Triton X-100 solution. To prepare PI standard solutions, 2.5 mM liver PI in 1% Triton X-100 is sequentially diluted with 1% Triton X-100 solution. The 25 mM liver PI in 10% Triton X-100 solution is stored at −20 °C.

#### 6.7.4. Procedure

1. Pipette each sample or PI standard solution (10 µL) into a 0.2-mL tube.
2. Add Reagent I1 (10 µL) to each tube, incubate at 37 °C for 60 min, and then heat at 96 °C for 3 min using a thermal cycler.
3. Precipitate the denatured enzyme by centrifugation (7200× *g*, 5 min, room temperature).
4. Pipette the supernatant (10 µL) into a 96-well black plate.
5. Add Reagent I2 (10 µL) to each well and incubate at 25 °C for 120 min.
6. Add Reagent I3 (80 µL) to each well and incubate at 45 °C for 60 min.
7. Add Amplex Red Stop Reagent (20 µL) to each well.
8. Measure the fluorescence intensity at 544 nm (excitation) and 590 nm (emission) using a microplate reader.

#### 6.7.5. Sensitivity and Specificity

The standard curve for the PI measurement is hyperbolic at concentrations below 500 μM [24]. The detection limit is 2 µM (20 pmol in the reaction mixture). There are no differences in the fluorescence changes in response to liver PI, soy PI, 1,2-dioleoyl PI, 1-palmitoyl-2-oleoyl PI, LPI, PI(4)P, and PI(5)P, indicating that this PI measurement is not affected by the chain length or the number of double bonds, and does not distinguish among PI, LPI, PI(4)P, and PI(5)P. On the other hand, PI(3)P, PI(3,4)P_2_, PI(3,5)P_2_, PI(4,5)P_2_, and PI(3,4,5)P_3_ exhibit no or negligible fluorescence increases. In this PI assay, PC, PE, PS, PA, PG, CL, and SM lead to no increase in fluorescence. In the recovery test using the cellular lipid extract, the mean recovery of liver PI in concentrations of 25–250 μM is 99.8% [24]. Excellent linearity of the PI assay is obtained when the cellular lipid extract is serially diluted [24].

### 6.8. Protocol for Enzymatic Fluorometric Measurement of PG + CL

#### 6.8.1. Strategy

There are five reaction steps for the enzymatic fluorometric measurement of PG + CL [23] (Figure 3f).

1. CL is hydrolyzed by PLD to PG and PA.
2. PG is hydrolyzed by PLD to glycerol and PA.
3. Glycerol is phosphorylated by glycerol kinase to G3P.
4. G3P is oxidized by G3P oxidase, generating H_2_O_2_ and dihydroxyacetone phosphate.
5. In the presence of peroxidase, H_2_O_2_ reacts with Amplex Red to produce resorufin.

#### 6.8.2. Materials

- PLD from *Streptomyces chromofuscus* (T-222, Asahi Kasei Pharma).
- Glycerol kinase from *Cellulomonas* sp. (GYK-301, Toyobo, Osaka, Japan).
- ATP (adenosine 5′-triphosphate disodium salt trihydrate) (018-16911, FUJIFILM Wako Pure Chemical).
- Heart CL (CL disodium salt from bovine heart) (840012P, Avanti Polar Lipids).
- Triton X-100 (see Section 6.2.1). Peroxidase, Amplex Red, Amplex Red Stop Reagent, and 96-well black flatbottom plate (see Section 6.2.1). G3P oxidase (see Section 6.6.2).

#### 6.8.3. Reagents

- Reagent L1: 5 U/mL PLD, 1.5 mM CaCl_2_, 50 mM NaCl, and 50 mM Tris-HCl (pH 7.4).
- Reagent L2: 5 U/mL glycerol kinase, 10 U/mL G3P oxidase, 5 U/ml peroxidase, 300 µM Amplex Red, 4.5 mM ATP, 2 mM MgCl_2_, 0.2% Triton X-100, 50 mM NaCl, and 50 mM Tris-HCl (pH 7.4).
- Solubilize heart CL (average M.W. 1494.32) at 25 mM in 10% Triton X-100 aqueous solution, and then dilute with water to 2.5 mM in 1% Triton X-100 solution. To prepare CL standard solutions, 2.5 mM heart CL in 1% Triton X-100 is sequentially diluted with 1% Triton X-100 solution. The 25 mM heart CL in 10% Triton X-100 solution is stored at −20 °C.

#### 6.8.4. Procedure

1. Pipette each sample or CL standard solution (10 µL) into a 96-well black plate.
2. Add Reagent L1 (40 µL) to each well and incubate at 37 °C for 30 min.
3. Add Reagent L2 (50 µL) to each well and incubate at room temperature for 30 min.
4. Add Amplex Red Stop Reagent (20 µL) to each well.
5. Measure the fluorescence intensity at 544 nm (excitation) and 590 nm (emission) using a microplate reader.

#### 6.8.5. Sensitivity and Specificity

The standard curve for CL measurement is linear at concentrations below 150 μM [23]. The detection limit is 1 µM (10 pmol in the reaction mixture). There are no differences in the fluorescence changes in response to heart CL, tetraoleoyl CL, egg PG, soy PG, 1-palmitoyl-2-oleoyl PG, and LPG, indicating that this measurement is not affected by the chain length or the number of double bonds, and does not distinguish among CL, PG, and LPG. Therefore, this assay quantifies the sum of PG and CL (PG + CL). In the recovery test using the cellular lipid extract, the mean recovery of heart CL in concentrations of 12.5–100 μM is 100.2% [23]. Excellent linearity of the PG + CL assay is obtained when the cellular lipid extract is serially diluted [23].

### 6.9. Protocol for Enzymatic Fluorometric Measurement of SM

#### 6.9.1. Strategy

There are four reaction steps for the enzymatic fluorometric measurement of SM [22] (Figure 3g).

1. SM is hydrolyzed by SMase to phosphocholine and ceramide.
2. Phosphocholine is dephosphorylated by alkaline phosphatase to choline.
3. Choline is oxidized by choline oxidase, generating two H_2_O_2_ molecules and betaine.
4. In the presence of peroxidase, H_2_O_2_ reacts with Amplex Red to produce resorufin.

#### 6.9.2. Materials

- SMase from *Bacillus cereus* (S9396, Sigma-Aldrich, St. Louis, MO, USA).
- Alkaline phosphatase from calf intestine (47785055, Oriental Yeast).
- Egg SM (SM from chicken egg) (860061P, Avanti Polar Lipids).
- Triton X-100 (see Section 6.2.1). Choline oxidase, peroxidase, Amplex Red, Amplex Red/UltraRed Stop Reagent, and 96-well black flatbottom plate (see Section 6.3.2).

#### 6.9.3. Reagents

- Reagent M1: 1 U/mL SMase, 20 U/mL alkaline phosphatase, 1.5 mM MgCl_2_, 50 mM NaCl, and 50 mM Tris-HCl (pH 7.4).
- Reagent M2: 4 U/mL choline oxidase, 5 U/mL peroxidase, 300 µM Amplex Red, 0.2% Triton X-100, 50 mM NaCl, and 50 mM Tris-HCl (pH 7.4).
- Solubilize egg SM (average M.W. 710.70) at 25 mM in 10% Triton X-100 aqueous solution, and then dilute with water to 2.5 mM in 1% Triton X-100 solution. To prepare SM standard solutions, 2.5 mM egg SM in 1% Triton X-100 is sequentially diluted with 1% Triton X-100 solution. The 25 mM egg SM in 10% Triton X-100 solution is stored at −20 °C.

#### 6.9.4. Procedure

1. Pipette each sample or SM standard solution (10 µL) into a 96-well black plate.
2. Add Reagent M1 (40 µL) to each well and incubate at 37 °C for 30 min.
3. Add Reagent M2 (50 µL) to each well and incubate at room temperature for 30 min.
4. Add Amplex Red Stop Reagent (20 µL) to each well.
5. Measure the fluorescence intensity at 544 nm (excitation) and 590 nm (emission) using a microplate reader.

#### 6.9.5. Sensitivity and Specificity

The standard curve for the SM measurement is quadratic at concentrations below 10 μM and linear between 10 and 100 μM [22]. The detection limit is 0.5 µM (5 pmol in the reaction mixture). There are no differences in the fluorescence changes in response to egg SM, brain SM, and palmitoyl SM, indicating that this SM measurement is not affected by the chain length or the number of double bonds. In this SM assay, other choline-containing phospholipids, SPC, PC, and LPC, lead to no or negligible increases in fluorescence. In the recovery test using the cellular lipid extract, the mean recovery of palmitoyl SM in concentrations of 12.5–50 μM is 100.3% [22]. Excellent linearity of the SM assay is obtained when the cellular lipid extract is serially diluted [22].

## 7. Future Directions

Our enzyme-based fluorometric methods enable simple and high-throughput, but not time-consuming, quantification of major phospholipid classes. All enzymes and compounds used in these assays are commercially available at present. These assays have high sensitivity and high accuracy (Table 1), and will be applied to cells, intracellular organelles, tissues, fluids, lipoproteins, and extracellular vesicles for elucidating physiological, pathological, and molecular mechanisms and for identifying disease biomarkers. 

These enzymatic fluorometric assays are more sensitive than the assays using TLC or HPLC. Mass spectrometry has extremely high sensitivity (femto-molar range) to detect phospholipid molecular species. However, it is difficult to quantify phospholipid classes using mass spectrometry. In addition, mass spectrometry takes a longer time to analyze many samples than the enzymatic assays. The combination of enzymatic fluorometric assays and mass spectrometry will make it possible to comprehensively characterize the phospholipid compositions in biological membranes. On the other hand, enzymatic fluorometric assays for measuring lysophospholipid classes still need to be developed.

## Figures and Tables

**Table 1:** Sensitivities and specificities of enzymatic fluorometric assays.

Assay	Detection Limit (pmol)	Detectable Phospholipid Class
PC assay	10	PC, plasmanylcholine
PE assay	10	PE, plasmenylethanolamine, LPE
PS assay	50	PS, LPS
PA assay	50	PA, LPA
PI assay	20	PI, LPI, PI(4)P, PI(5)P
PG + CL assay	10	PG, CL, LPG
SM assay	5	SM

## Abbreviations

4-AA	4-amino antipyrine
ABC	ATP-binding cassette
CL	cardiolipin
DAOS	*N*-ethyl-*N*-(2-hydroxy-3-sulfopropyl)-3,5-dimethoxyaniline
ER	endoplasmic reticulum
G3P	glycerol-3-phosphate
GPL	glycerophospholipid
GPL-PLD	glycerophospholipid-specific phospholipase D
HDL	high density lipoprotein
HPLC	high-performance liquid chromatography
HPPA	3-(4-hydroxyphenyl)propionic acid
LDL	low density lipoprotein
LPA	lysophosphatidic acid
LPC	lysophosphatidylcholine
LPE	lysophosphatidylethanolamine
LPG	lysophosphatidylglycerol
LPI	lysophosphatidylinositol
LPS	lysophosphatidylserine
Lp-X	lipoprotein-X
PA	phosphatidic acid
PC	phosphatidylcholine
PE	phosphatidylethanolamine
PG	phosphatidylglycerol
PI	phosphatidylinositol
PLD	phospholipase D
PS	phosphatidylserine
SM	sphingomyelin
SMase	sphingomyelinase
SPC	sphingosylphosphorylcholine
TLC	thin-layer chromatography
TOOS	*N*-ethyl-*N*-(2-hydroxy-3-sulfopropyl)-3-methylaniline
VLDL	very low density lipoprotein
